# Uric Acid to High-Density Lipoprotein Cholesterol Ratio is a Novel Marker to Predict Functionally Significant Coronary Artery Stenosis

**DOI:** 10.1155/2022/9057832

**Published:** 2022-10-13

**Authors:** Fanqi Li, Donghui Zhao, Qiuyu Li, Xiaolong Lin, Haoxuan Sun, Qian Fan

**Affiliations:** Department of Cardiology, Beijing an Zhen Hospital, Capital Medical University, and Beijing Institute of Heart, Lung, and Blood Vessel Disease, Beijing, China

## Abstract

**Background:**

Intermediate coronary stenosis (ICS) is defined as a visually estimated percentage of diameter stenosis ranging between 40% and 70% by conventional coronary angiography (CAG). Whether to perform percutaneous coronary intervention (PCI) for these lesions is a challenge in clinical practice. The fractional flow reserve (FFR) can guide treatment by determining the functional significance of ICS. Studies have shown that some clinical indicators can be used to predict FFR. However, there is little research on this in the Chinese population.

**Methods:**

We retrospectively analyzed 690 patients who underwent FFR measurements to determine the functional significance of a single ICS. Patients were divided into 2 groups: FFR ≤0.8 (*n* = 280) and FFR >0.8 (*n* = 410). We compared the clinical factors between the two groups and performed multivariate logistic regression analyses to explore the risk factors. In addition, receiver-operating characteristic (ROC) curves were constructed for FFR ≤0.8 diagnoses.

**Results:**

The mean UHR (uric acid to high-density lipoprotein cholesterol ratio) level was significantly higher in the FFR ≤0.8 group (*p* < 0.001). UHR corrects negatively with FFR (*r* = −0.44, *p* < 0.001). High-level UHR was an independent risk factor for the FFR ≤0.8 (OR = 7.17, 95% CI 4.17–12.34). The area under the curve (AUC) of the UHR diagnostic capacity for the FFR ≤0.8 is 0.77, with 77.3% sensitivity and 68.2% specificity.

**Conclusion:**

UHR levels were significantly increased in patients with hemodynamically significant coronary lesions. UHR is a novel predictor of functionally significant lesions in patients with a single-vessel disease of ICS.

## 1. Introduction

The incidence of coronary artery disease (CAD) has markedly increased in the past 20–30 years in China [1]. In clinical practice, CAG is a widely used method to assess the severity and extent of CAD. However, because CAG only involves anatomic factors, it will be powerless for ICS [2]. Whether to perform PCI for ICS is a challenge for cardiologists. FFR solved this problem from the viewpoint of functional significance by measuring the distal coronary artery pressure and aortic pressure [3]. Nowadays, FFR is widely regarded as the gold standard to guide treatment for ICS [4]. FFR values ≤ 0.8 indicate coronary stenosis associated with functional significance [5]. However, FFR has not been widely used in the diagnosis and treatment in clinical practice due to the extra operation time, cost, and use of adenosine during examination [6]. Hence, finding a reliable predictive biomarker would be welcomed.

Fortunately, several clinical studies have been performed that show finding an indicator to predict FFR is feasible. Erdoğan et al. suggest that the systemic immune-inflammation index, calculated by neutrophil *∗* platelets/lymphocytes, can predict FFR ≤0.80, with 78.4% sensitivity and 64.0% specificity [7]. In addition, uric acid (UA) and some biomarkers of lipid modification also showed satisfactory forecasting ability for FFR [8, 9]. Nevertheless, studies on predicting FFR in the Chinese population are rare. Considering factors (such as white blood cells [10] and high-density lipoprotein [11]) affected by race, region, and diet, additional clinical studies from different countries are essential.

As we all know, UA and high-density lipoprotein cholesterol (HDL-C) disorders are both considered as risk factors for CAD. Previous studies showed that UHR is associated with hypertension control [12], hepatic steatosis [13], thyroiditis [14], and cardiovascular mortality [15]. Some mechanistic studies reveal that high-level UA and low HDL-C may exert synergistic deleterious effects on the cardiovascular system by increasing endothelial oxidative damage and insulin resistance [16–19]. Therefore, we speculated that a combination of serum UA and HDL-C could be a better biomarker of the severity of CVD. To the best of our knowledge, the association between UHR and FFR has not been explored.

In this present study, we aim to investigate the relationship between UHR and FFR and find indicators to predict the functional significance for ICS in the Chinese population.

## 2. Methods

### 2.1. Study Cohort

**Figure 1:**
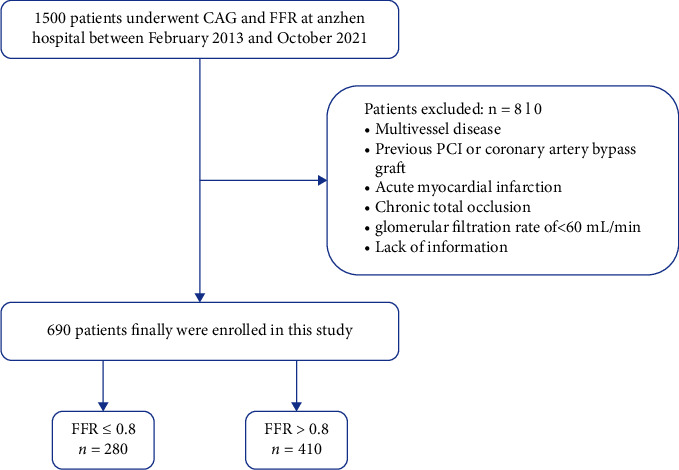
Population flow chart of enrolled patients.

In this observational retrospective study, 1500 inpatients with ICS discovered by CAG underwent FFR from February 2013 to October 2021 at the Anzhen Hospital (Beijing, China) and were consecutively enrolled in this study. Patients were eligible if they fulfilled the following inclusion criteria: (1) a single angiographically intermediate lesion (40%–70% stenosis by visual assessment, defined as intermediate lesions [20]) in a native coronary artery with a reference diameter of more than 2.5 mm; and (2) performed an FFR exam. An FFR ≤0.8 was described as hemodynamically significant. Exclusion criteria were the presence of multivessel disease, previous PCI or coronary artery bypass graft, acute myocardial infarction, chronic total occlusion, a glomerular filtration rate of <60 mL/min, and a lack of information. Finally, 690 patients were enrolled, and patients were assigned to the FFR ≤0.8 (*n* = 280) group or the FFR >0.8 (*n* = 410) group. The flow chart of the selection process is shown in Figure 1.

### 2.2. Procedure

Demographic features and laboratory data were collected for all participants from the hospital information system. All patients underwent blood sampling and laboratory tests in the early morning after admission on an empty stomach. The diagnostic criteria for hypertension and diabetes mellitus were based on authoritative international guidelines. Smoking was defined as a history of smoking in the previous 6 months before admission. Multivessel disease was defined as stenosis (≥40% diameter stenosis) in at least two major epicardial coronary arteries. Based on the result of FFR and patients' conditions, at least two cardiologists are involved in clinical treatment strategies.

### 2.3. CAG and Fractional Flow Reserve

Both CAG and FFR were performed according to clinical standards. Intermediate coronary stenosis was defined as a coronary lesion with a visually estimated percentage diameter stenosis ranging between 40% and 70% of a major epicardial vessel. The CAG results were independently evaluated by two experienced interventional cardiologists who were blinded to this study. An FFR was carried out with the QUANTIEN platform (St. Jude Medical, St. Paul, MN, USA). A pressure wire (Aeris, St. Jude Medical) was advanced distal to the stenosis. After the intravenous administration of 140 mg/kg/min adenosine, we obtained distal coronary artery pressure by pressure wire and aortic pressure by guiding catheter. The ratio of the two pressures is FFR.

### 2.4. Statistical Analysis

SPSS 22.0 (SPSS Inc., IL, USA) was used for statistical testing. Histograms and analytical Kolmogorov–Smirnov test methods to determine whether variables were normally distributed. The data of normal distribution were expressed as average value ± standard deviation, and Student's *t*-tests were used for comparison between groups. M (P25, P75) was used for measurement data that did not conform to a normal distribution, and the Mann–Whitney *U* test was used for comparison between groups. Categorical variables were presented as numbers and percentages and were compared using the *χ*^2^-test or Fisher's exact test. The receiver-operating characteristic (ROC) curves were constructed, and the AUCs were calculated to obtain the cutoff values. Variables that might be a possible confounding factor for the functionally significant stenosis, such as age, gender, hypertension, diabetes mellitus, smoking, white blood cells, monocytes, LDL-C, red blood cell distribution width, total bile acid, BMI ≥24, and UHR, were included in the univariate analyses. The variables which were determined as *p* < 0.1 in univariate analyses and some classic risk factors were included in a multivariate logistic regression analysis. To avoid multicollinearity, we did not include neutrophils, urea, and HDL-C in the regression models. A two-sided *p*-value <.05 was considered statistically significant. In addition, MedCalc (version 20.0.22) was used for the comparison of AUCs.

## 3. Results

### 3.1. Baseline Demographic and Clinical Characteristics

Baseline characteristic features are shown in Table 1. There was no difference between the two groups regarding age, heart rate, systolic blood pressure, and prevalence of diabetes mellitus, hypertension, and smoking status. Ejection fraction, white blood cell, red blood cell, platelet, monocyte, lymphocyte, hemoglobin, hematocrit, triglyceride, total cholesterol, low-density lipoprotein cholesterol, HDL-C, fasting glucose, glycosylated hemoglobin, C-reactive protein, and medicine were similar between the groups. The proportion of males, body mass index (BMI), neutrophils, total bile acid, urea, and creatinine were higher in the FFR ≤0.8 group (*p* < 0.05, Table 1). UA and UHR were significantly higher in the FFR ≤0.8 group, whereas HDL-C was lower (*p* < 0.001, Table 1).

### 3.2. Correlations of the UHR with Biochemical Parameters

To further explore the associations between the UHR, FFR, and biochemical parameters, we analyzed an array of correlations. The BMI (*r* = 0.278, *p* < 0.001), hemoglobin (*r* = 0.221, *p* < 0.001), triglyceride (*r* = 0.249, *p* < 0.001), urea (*r* = 0.211, *p* < 0.001), creatinine (*r* = 0.250, *p* < 0.001), and homocysteine (*r* = 0.224, *p* < 0.001) showed significant positive correlations with the UHR (Table 2). In addition, the UHR showed a significant negative correlation with the FFR (*r* = −0.436, *p* < 0.001, Table 2).

### 3.3. Univariate and Multivariate Logistic Regression Analyses

We performed logistic regression analyses with two separate models according to the continuous and categorical values of UHR. Hosmer and Lemeshow tests for the models are 0.389 and 0.599. After adjusting for confounding factors, UHR is the only independent predictor (Table 3). UHR >310.8 (cutoff value) was independently associated with an FFR ≤0.8 (OR = 7.171, 95% CI 4.168–12.338, *p* < 0.001, Table 3).

### 3.4. ROC Curve Analysis

**Figure 2:**
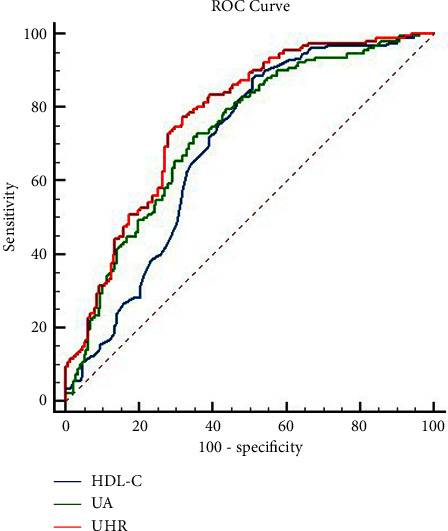
ROC curves.

To investigate and compare the predictive capacity of UA, HDL-C, and UHR, ROC curves were made (Figure 2). An AUC value of 0.728 (95%CI: 0.673–0.782, *p* < 0.001) with a positive likelihood ratio of 2.08 for the UA and an AUC value of 0.692 (95%CI: 0.635–0.749, *p* < 0.001) with a positive likelihood ratio of 1.73 for HDL-C. The AUC of UHR was 0.770 (95% CI: 0.721–0.815, *p* < 0.001), and the optimal cutoff value was 310.8, with a Youden index of 0.455. The sensitivity of the UHR for the diagnosis of the FFR ≤0.8 was 77.3%, the specificity was 68.2%, and the positive and negative likelihood ratios were 2.43 and 0.33, respectively. The AUC for UHR is 0.043 larger than UA (*Z* = 1.998, *p* < 0.05) and is 0.078 larger than HDL-C (*Z* = 3.699, *p* < 0.001, Table 4).

## 4. Discussion

In this study, we demonstrated significantly higher UHR values in the FFR ≤0.8 group. The UHR was negatively correlated with FFR and showed significant associations with clinical parameters such as BMI, creatinine, and triglyceride. Furthermore, the UHR, as a novel biomarker combining UA and HDL-C, showed excellent diagnostic capability for functionally significant stenosis in patients with a single vessel disease of intermediate stenosis. To the best of our knowledge, this is the first study to explore the association between the UHR and ICS.

Despite interventional technology advances, accurate assessment of ICS remains difficult in the catheterization laboratory [20]. Whether to perform PCI for these lesions is a challenge in clinical practice. FFR solved this problem by measuring the distal coronary artery pressure and aortic pressure. FFR values ≤ 0.8 indicate coronary stenosis associated with functional significance [21]. However, FFR has not been widely used in the diagnosis and treatment in clinical practice due to the extra operation time, cost, and use of adenosine during examination [6]. Hence, finding a reliable predictive biomarker would be welcomed. In order to avoid the interference of some confounding factors, we designed a well-defined patient population with a single vessel disease.

Research on the prediction of FFR has always been a hot issue in the cardiovascular field. On the one hand, some other functional indicators based on FFR are developing rapidly, such as instant wave-free ratio (iFR), coronary CT angiogram FFR (FFR_CT_), and quantitative coronary angiography FFR (QFR) [22]. These emerging indicators have the advantages of simplicity, time savings, and no need to use adenosine. On the other hand, based on diameter stenosis determined by CAG, several clinical risk factors were also taken into account and significantly improved predictive accuracy [23]. Erdoğan *M* et al. suggested a systemic immune-inflammation index, calculated by neutrophil  ^*∗*^ platelets/lymphocytes, can predict the FFR ≤0.8, with 78.4% sensitivity and 64.0% specificity [7]. In addition, UA and some biomarkers of lipid modification also showed satisfactory forecasting ability for FFR [8, 9]. It is worth noting that all the participants in these three studies [7–9] were patients with a single-vessel coronary artery stenosis, like our study design. Considering factors such as white blood cells [24] and HDL [11] affected by race, region, and diet, additional clinical studies from different countries are essential. Besides, studies on predicting FFR are rare in China. In the present study, we demonstrated the predictive power of UHR in the Chinese population.

Serum uric acid is the final product of purine nucleotide metabolism and is widely regarded as a risk factor for CHD. Previous clinical studies showed that a high UA level is associated with CHD as well as its severity and prognosis [25, 26]. Salih et al. also showed that UA level is significantly higher in the hemodynamically significant lesions group detected by FFR [8]. At the molecular level, uric acid leads to endothelial dysfunction by enhancing endothelial nitric oxide synthase phosphorylation and mediating endoplasmic reticulum stress [27]. When the intracellular environment changes, UA converts into a pro-oxidant agent to accelerate the progression of CAD [28]. These clinical studies and pathophysiological mechanisms of UA support our results.

HDL-C is a highly heterogeneous polymer composed of hundreds of proteins and lipids. In brief, it acts as an antiatherosclerosis agent by transporting cholesterol from tissues and cells outside the liver to the liver [29]. In the Framingham Heart Study and the Prospective Cardiovascular Munster Study, researchers found an increase of 1 mg/dl (0.026 mmol/L) in the HDL-C level was associated with a 2%–3% reduction in the risk of cardiovascular disease [30]. Xue Tian et al. proved that HDL was an independent protective factor for FFR reduction in 296 UA patients [31]. Our study found similar results in patients with a single vessel disease of intermediate stenosis.

As discussed above, the relationship between UA, HDL-C, and CAD has been widely accepted. Some basic experimental studies have shown that UA and HDL-C may interact with each other to exacerbate the progression of cardiovascular disease by damaging endothelial cell function and enhancing oxidative stress [16–19]. In addition, clinical studies have demonstrated the relationship of UHR with metabolic syndrome [32], diabetes control [33], and cardiovascular mortality in patients on peritoneal dialysis [15]. Hence, we speculated that a combination of serum UA and HDL-C could be a better biomarker of the severity of CVD. Our results demonstrated that the UHR, as a novel marker, showed better prediction power than UA or HDL-C for the FFR ≤0.8 in patients with a single vessel disease of intermediate stenosis.

### 4.1. Study Limitations

Our study has some limitations. First, the single-center nature of this study and the relatively small number of enrolled patients may have introduced selection bias. Second, because this study only investigated the hemodynamic significance of a single vessel disease, further studies are needed to determine whether our conclusions are applicable to other patients, such as those with multivessel disease and previous PCI.

## 5. Conclusion

In conclusion, our study suggest that UHR is independently associated with the FFR ≤0.8 and could predict functionally significant lesions in Chinese patients with a single vessel disease of intermediate stenosis.

## Figures and Tables

**Table 1:** Baseline demographic and clinical characteristics.

Factor	FFR ≤ 0.8 (*n* = 280)	FFR > 0.8 (*n* = 410)	t/*χ*^2^/*Z*	*p*
Age (years)	57.84 ± 9.78	58.91 ± 8.99	−1.034	0.302
Male (*n*(%))	208 (74.3%)	270 (65.9%)	5.558	0.018
BMI (kg/m^2^)	25.79 ± 2.77	25.11 ± 2.71	2.238	0.026
Hypertension (*n*(%))	159 (56.8%)	212 (51.7%)	1.726	0.189
DM (*n*(%))	86 (30.7%)	105 (25.6%)	2.166	0.141
Smoking (*n*(%))	88 (31.4%)	124 (30.2%)	0.110	0.740
HR (bpm)	69.85 ± 6.95	70.01 ± 6.38	−0.224	0.823
SBP (mmHg)	129.02 ± 8.90	127.95 ± 6.98	1.139	0.256
EF (%)	64.82 ± 4.78	65.14 ± 4.14	−0.650	0.516
WBC (10^12^/L)	6.30 (5.41,7.66)	6.06 (5.05,7.05)	−1.932	0.053
RBC (10^12^/L)	4.63 ± 0.45	4.61 ± 0.42	0.517	0.605
PLT (10^9^/L)	217.57 ± 45.27	220.31 ± 48.02	−0.529	0.597
MONO (10^9^/L)	0.34 (0.27,0.45)	0.33 (0.25,0.42)	−1.660	0.097
LYM (10^9^/L)	1.77 (1.42,2.15)	1.77 (1.38,2.14)	−0.363	0.717
NE (10^9^/L)	4.01 (3.25,5.02)	3.71 (2.96,4.63)	−2.145	0.032
HG (g/L)	142.73 ± 14.67	141.38 ± 13.95	0.857	0.392
HCT (%)	41.20 ± 3.72	40.97 ± 3.61	0.568	0.570
MCV (fl)	89.04 ± 3.79	89.15 ± 4.17	−0.233	0.816
MCH (pg)	30.85 ± 1.62	30.73 ± 1.60	0.704	0.482
MCHC (g/L)	346.18 ± 10.69	344.37 ± 10.16	1.563	0.119
RDW (fl)	42.19 ± 2.55	42.14 ± 2.51	0.168	0.867
TG (mmol/L)	1.40 (0.99,1.95)	1.34 (0.93,1.81)	−1.161	0.246
TC (mmol/L)	4.07 (3.4,4.75)	4.03 (3.43,4.90)	−0.023	0.981
LDL-c (mmol/L)	2.38 (1.73,2.97)	2.34 (1.77,2.83)	−0.521	0.602
HDL-c (mmol/L)	1.04 (0.92,1.14)	1.19 (1.00,1.39)	−6.006	<0.001
Glu (mmol/L)	5.82 (5.27,6.81)	5.6 (5.17,6.45)	−1.505	0.132
HbA1c (mmol/L)	5.9 (5.6,6.6)	5.8 (5.4,6.5)	−1.384	0.166
ALT(mmol/L)	22 (16,33)	21 (15,30)	−1.269	0.204
AST (mmol/L)	21.5 (18,26)	21 (18,26)	−0.722	0.470
TP (g/L)	69.64 ± 5.48	69.31 ± 5.71	0.540	0.590
ALB (g/L)	43.41 ± 3.91	43.29 ± 3.95	0.275	0.783
T-bil (*μ*mol/L)	13.2 (10.4,16.8)	13.2 (10.5,16.5)	−0.026	0.979
D-bil (*μ*mol/L)	2.74 (2.04,3.52)	2.87 (2.13,3.79)	−0.338	0.735
TBA (*μ*mol/L)	2.7 (1.4,4.5)	2.2 (1.3,3.6)	−2.031	0.042
ChE(KU/L)	8.2 (7.4,9.3)	8.3 (7.2,9.2)	−0.03	0.976
GGT (U/L)	26 (19,41)	26 (18,39)	−0.818	0.413
ALP (U/L)	77 (62,90)	78 (66,93)	−1.022	0.307
Urea (mmol/L)	5.2 (4.5,6.2)	5.0 (4.3,5.8)	−2.070	0.038
UA (*μ*mol/L)	366.7 (324.8,408.6)	310.8 (277.1,358.8)	−7.113	<0.001
Cr (*μ*mol/L)	69.73 ± 13.20	65.34 ± 12.16	3.134	0.002
Hcy (*μ*mol/L)	11.7 (9.3,15.8)	11.9 (8.9,14.8)	−0.591	0.554
CRP (mg/L)	1.00 (0.50,2.08)	0.85 (0.39,2.09)	−0.942	0.346
UHR	365.3 (314.6,422.6)	268.8 (199.2,345.4)	−8.445	<0.001
FFR	0.75 (0.70,0.78)	0.87 (0.84,0.91)	−15.636	<0.001

Angiography				
LM	8 (2.9%)	12 (2.9%)	0.003	0.957
LAD	230 (82.1%)	321 (78.3%)	1.533	0.261
LCX	13 (4.6%)	25 (6.1%)	0.677	0.411
RCA	29 (10.4%)	52 (12.7%)	0.869	0.351

Medicine				
Aspirin (*n*(%))	270 (96.4%)	387 (94.4%)	1.518	0.218
Statin (*n*(%))	255 (91.1%)	368 (89.8%)	0.328	0.567
UA-lowering drugs (*n*(%))	16 (5.7%)	18 (4.4%)	0.623	0.430
Beta blocker (*n*(%))	171 (61.1%)	240 (58.5%)	0.444	0.505

Abbreviations: BMI, body mass index; DM, diabetes mellitus; HR, heart rate; SBP, systolic blood pressure; EF, ejection fraction; WBC, white blood cell; RBC, red blood cell; PLT, platelet; MONO, monocyte; LYM, lymphocyte; NE, neutrophil; HG, hemoglobin; HCT, hematocrit; MCH, mean corpuscular hemoglobin; MCHC, mean corpuscular-hemoglobin concentration; MCV, mean corpuscular volume; RDW, red blood cell distribution width; TG, triglyceride; TC, total cholesterol; LDL-C, low-density lipoprotein cholesterol; HDL-C, high-density lipoprotein cholesterol; GLu, glucose; HbA1c, glycosylated hemoglobin; ALT, alanine aminotransferase; AST, aspartate aminotransferase; TP, total protein; ALB, albumin; T-Bil, total bilirubin, D-Bil, direct bilirubin; TBA, total bile acid; ChE, cholinesterase; GGT, glutamyl transpeptidase; ALP, alkaline phosphatase; UA, uric acid; Cr, creatinine; Hcy, homocysteine; CRP, C-reactive protein; LM, left main coronary artery; LAD, left anterior descending; LCX, left circumflex; RCA, right coronary artery.

**Table 2:** Correlations of the UHR with biochemical parameters.

	Age	BMI	HR	SBP	EF	WBC	RBC	PLT	MONO	TC	LDL-C	Glu	HbA1c	ALT	AST	TP	ALB	T-bil
UHR	−0.086	0.278 ^*∗∗*^	−0.019	−0.008	−0.048	0.139 ^*∗*^	0.198 ^*∗*^ ^*∗*^	−0.104	0.187 ^*∗*^ ^*∗*^	−0.131 ^*∗*^	−0.031	0.077	0.085	0.156 ^*∗*^ ^*∗*^	0.092	0.001	−0.058	0.123 ^*∗*^
	0.044	0.148 ^*∗*^ ^*∗*^	0.221 ^*∗*^ ^*∗*^	0.178 ^*∗*^ ^*∗*^	−0.045	0.086	0.194 ^*∗*^ ^*∗*^	−0.008	0.249 ^*∗*^ ^*∗*^	0.066	0.044	0.017	−0.080	0.211 ^*∗*^ ^*∗*^	0.250 ^*∗*^ ^*∗*^	0.244 ^*∗*^ ^*∗*^	0.153 ^*∗*^ ^*∗*^	−0.436 ^*∗*^ ^*∗*^
	LYM	NE	HG	HCT	MCV	MCH	MCHC	SDW	TG	D-bil	TBA	ChE	ALP	Urea	Cr	Hcy	CRP	FFR

^*∗*^*p* < 0.05,  ^*∗∗*^*p* < 0.01. Abbreviations: BMI, body mass index; DM, diabetes mellitus; HR, heart rate; SBP, systolic blood pressure; EF, ejection fraction; WBC, white blood cell; RBC, red blood cell; PLT, platelet; MONO, monocyte; LYM, lymphocyte; NE, neutrophil; HG, hemoglobin; HCT, hematocrit; MCH, mean corpuscular hemoglobin; MCHC, mean corpuscular-hemoglobin concentration; MCV, mean corpuscular volume; RDW, red blood cell distribution width; TG, triglyceride; TC, total cholesterol; LDL-C, low-density lipoprotein cholesterol; HDL-C, high-density lipoprotein cholesterol; GLu, glucose; HbA1c, glycosylated hemoglobin; ALT, alanine aminotransferase; AST, aspartate aminotransferase; TP, total protein; ALB, albumin; T-Bil, total bilirubin, D-Bil, direct bilirubin; TBA, total bile acid; ChE, cholinesterase; GGT, glutamyl transpeptidase; ALP, alkaline phosphatase; UA, uric acid; Cr, creatinine; Hcy, homocysteine; CRP, C-reactive protein; LM, left main coronary artery; LAD, left anterior descending; LCX, left circumflex; RCA, right coronary artery.

**Table 3:** Multivariate logistic regression analyses.

Variables	Univariate		Multivariate	
	Or (95% CI)	*p*-value	Or (95% CI)	*p*-value
Age	0.988 (0.965–1.011)	0.301	—	
Male	1.498 (1.070–2.098)	0.019	1.166 (0.596–2.282)	0.653
Hypertension	1.227 (0.904–1.666)	0.189	—	
DM	1.288 (0.919–1.804)	0.142	1.437 (0.802–2.575)	0.223
Smoking	1.057 (0.761–1.469)	0.740	0.573 (0.303–1.082)	0.086
WBC	1.117 (0.992–1.257)	0.054	1.153 (0.935–1.423)	0.184
MONO	4.352 (0.859–22.063)	0.097	0.391 (0.035–4.334)	0.445
RDW	1.007 (0.924–1.098)	0.866	—	
LDL-C	1.067 (0.825–1.381)	0.601	1.244 (0.906–1.722)	0.174
TBA	1.086 (0.998–1.182)	0.055	1.098 (0.980–1.230)	0.107
BMI ≥24	1.133 (0.713–1.800)	0.598	0.614 (0.351–1.075)	0.088
UHR (continuous variable)	1.011 (1.008–1.014)	<0.001	1.012 (1.009–1.015)	<0.001
Age	0.988 (0.965–1.011)	0.301	—	
Male	1.498 (1.070–2.098)	0.019	1.229 (0.631–2.393)	0.544
Hypertension	1.227 (0.904–1.666)	0.189	—	
DM	1.288 (0.919–1.804)	0.142	1.324 (0.745–2.352)	0.339
Smoking	1.057 (0.761–1.469)	0.740	0.700 (0.375–1.305)	0.262
WBC	1.117 (0.992–1.257)	0.054	1.137 (0.924–1.400)	0.224
MONO	4.352 (0.859–22.063)	0.097	0.624 (0.058–6.686)	0.697
RDW	1.007 (0.924–1.098)	0.866	—	
LDL-C	1.067 (0.825–1.381)	0.601	1.165 (0.853–1.592)	0.336
TBA	1.086 (0.998–1.182)	0.055	1.092 (0.975–1.224)	0.127
BMI ≥24	1.133 (0.713–1.800)	0.598	0.744 (0.430–1.288)	0.291
UHR (categorical variable)	7.118 (4.343–11.665)	<0.001	7.171 (4.168–12.338)	<0.001

Abbreviations: DM, diabetes mellitus; WBC, white blood cell; MONO, monocyte; LDL-C, low-density lipoprotein cholesterol; TBA, total bile acid; BMI, body mass index; UHR, UA to HDL-C ratio.

**Table 4:** Analysis of the AUCs.

	UHR	UA	HDL-C
Cutoff value	310.8	331.8	1.21
Sensitivity	77.33%	72.00%	88.67%
Specificity	68.16%	65.36%	48.60%
AUC	0.770	0.728	0.692
Difference with UHR	—	0.043	0.078
*p*-value	—	0.046	<0.001

Abbreviations: UA, uric acid; HDL-C, low-density lipoprotein cholesterol; UHR, UA to HDL-C ratio; AUC, the area under the curve.

## Data Availability

The data used to support the findings of this study are available from the corresponding author upon request.
